# Narcissistic Leaders and Their Victims: Followers Low on Self-Esteem and Low on Core Self-Evaluations Suffer Most

**DOI:** 10.3389/fpsyg.2018.00422

**Published:** 2018-03-29

**Authors:** Barbara Nevicka, Annebel H. B. De Hoogh, Deanne N. Den Hartog, Frank D. Belschak

**Affiliations:** ^1^Department of Psychology, University of Amsterdam, Amsterdam, Netherlands; ^2^Amsterdam Business School, University of Amsterdam, Amsterdam, Netherlands

**Keywords:** leader narcissism, abusive supervision, follower self-esteem, follower core self-evaluations, performance, exhaustion

## Abstract

Narcissistic leaders are self-absorbed and hold beliefs of entitlement and superiority. Their aggressive tendencies in the face of criticism and inclinations to validate their self-worth by derogating others may lead others to perceive them as being abusive. Here, we test the relationship between leader narcissism and followers’ perceptions of abusive supervision. Drawing upon research related to the behavioral plasticity hypothesis, we propose that followers with low self-esteem will perceive narcissistic leaders as more abusive than those with high self-esteem. Followers low on self-esteem are more insecure, more in need of approval from their supervisor and are more likely to interpret the haughty, derogatory attitude of narcissistic leaders as abusive. Such followers also make for ‘easier targets’ and thus may actually suffer more abusive behavior from their narcissistic leaders. In a first multi-source study of 85 leaders and 128 followers, we found support for the moderating role of follower self-esteem in the relationship between leader narcissism and perceived abusive supervision: Narcissistic leaders were rated as more abusive by followers who were low on self-esteem, but not those higher on self-esteem. In a second multi-source field study among 177 leader-follower dyads, we tested a moderated mediation model and showed that this finding also holds for the broader concept of follower core self-evaluations as a moderator. Abusive supervision, in turn, was related to lower follower performance and followers experiencing more burnout symptoms. Thus, followers low on self-esteem or low on core self-evaluations seem to suffer most from narcissistic leaders as they perceive them to be abusive and, in turn, these followers show reduced performance and more burnout symptoms when working for such leaders. This research thus identifies an important moderator that might help reconcile previous inconsistent findings regarding perceptions of narcissistic leaders.

## Introduction

Narcissism, a personality trait characterized by grandiose and overly positive self-views, is not only rising in Western individualistic countries (Twenge et al., 2008; Twenge and Foster, 2010), but also appears to be societally valued as evidenced by narcissists’ emergence as leaders (Brunell et al., 2008; Nevicka et al., 2011a; Grijalva et al., 2015a). The reason for this is that narcissistic individuals possess many characteristics that people associate with a prototypical leader (e.g., confidence, extraversion, dominance; Smith and Foti, 1998; Judge et al., 2002; Kellett et al., 2006; Paunonen et al., 2006). Furthermore, narcissists’ charm, humor, enthusiasm and often attractive charismatic vision (Galvin et al., 2010; Goncalo et al., 2010) engender positive first impressions (Back et al., 2010), which can facilitate successful appraisal in selection contexts and help narcissists rise to power.

The problem with narcissists’ rise to power, however, is that narcissists also have many negative interpersonal characteristics, such as a lack of empathy, exploitativeness, a sense of entitlement, antagonism and egocentrism (Sedikides and Campbell, 2017), which could lead them to abuse their power and adversely impact those they lead. For instance, narcissists are known to aggress against and derogate others when their ego is threatened (Bushman and Baumeister, 1998), and even sometimes aggress without provocation (Martinez et al., 2008; Lobbestael et al., 2014; Park and Colvin, 2015). Furthermore, they externalize blame while accepting credit for others’ success (Stucke, 2003), they are exceedingly critical of others and expect perfection (Stoeber et al., 2015), and they show unethical behavior (Soyer et al., 1999; Penney and Spector, 2002; Watts et al., 2013). Scholars have theorized that narcissists’ tendencies to act in a self-interested and dominant manner might predispose them to engage in abusive or destructive behavior as leaders (Tepper, 2007; Krasikova et al., 2013; Martinko et al., 2013). Interestingly, a recent study in an organizational setting found no direct relationship between leader narcissism and abusive supervision (Wisse and Sleebos, 2016), defined as sustained display of hostile verbal and non-verbal behaviors, excluding physical contact (Tepper, 2000). We propose that the extent to which narcissistic leaders are perceived as abusive depends on followers’ personality. What is interpreted as abusive behavior often substantially varies between individual perceivers (Tepper, 2000). Thus, we propose that while some followers may perceive narcissistic individuals in a leadership role as abusive, others may not.

In line with this proposition of differential perceptions of narcissistic leaders by different followers, findings regarding followers’ general perceptions of narcissistic leaders are mixed. Some studies show that followers had favorable perceptions of narcissistic leaders (Judge et al., 2006; Nevicka et al., 2011b; Owens et al., 2015), while others show followers having negative perceptions (Judge et al., 2006; Martin et al., 2016) and a recent meta-analysis showed no linear relationship between narcissism of leaders and perceptions of leader effectiveness (Grijalva et al., 2015a). These inconsistent findings suggest that moderators may play an important role in followers’ perceptions of narcissistic leaders. For instance, prior research shows that perceptions of narcissistic individuals in peer groups vary according to the length of acquaintance because the passage of time exposes narcissists’ negative characteristics. Thus, short-term acquaintances tend to evaluate narcissistic peers more positively, whereas over time with longer acquaintance these positive perceptions diminish (Carlson et al., 2011; Leckelt et al., 2015; Ong et al., 2016).

In a similar vein, followers with certain personality traits might be more sensitive to the toxic characteristics of narcissistic leaders, while others may be better able to cope with such leaders. Therefore, the current research set out to answer the important question of which followers would be most likely negatively impacted by narcissistic leaders? Specifically, we expect that narcissistic leaders will be perceived as abusive especially by followers with low self-esteem. By focusing on followers’ self-esteem as an important moderator, we thus help reconcile inconsistent findings regarding followers’ perceptions of narcissistic leaders.

### Leader Narcissism and Follower Self-Esteem

Self-esteem - the appraisal of a person’s self-worth (Leary and Baumeister, 2000) – has been theorized to be a personality trait which increases individuals’ susceptibility to leaders’ toxicity (Padilla et al., 2007; Thoroughgood et al., 2012). This suggests that self-esteem may moderate how followers perceive destructive leaders such as narcissistic leaders. We propose two main theoretical reasons why followers with low self-esteem (rather than high self-esteem) would perceive leaders as more abusive the more narcissistic they are, namely because of followers’: (1) greater *sensitivity* to narcissistic leaders’ negative characteristics and (2) greater likelihood to actually *encounter* narcissists’ abusive behavior.

Firstly, behavioral plasticity hypothesis contends that self-esteem moderates the extent to which individuals react to external cues (Brockner, 1988). Because they are uncertain of the appropriateness of their attitudes and behavior, individuals with low self-esteem are more sensitive and reactive to external social cues. In the organizational context, a leader would constitute an important contextual cue as the leader provides direction, evaluates the employee and has the power to reward or punish. Low self-esteem followers are therefore likely to be more perceptive of external cues such as their leader’s traits, than followers high on self-esteem (Elangovan and Xie, 1999; Avey et al., 2011).

In addition, low self-esteem individuals are more likely to interpret leaders’ toxic characteristics as stressful and threatening and they would be less able to cope with them (Smith and Petty, 1995). This does not mean that high self-esteem individuals would be completely oblivious to the toxic side of narcissistic individuals. Rather, they would be better equipped to deal with such leaders because of their better coping strategies in general, rely less on their leaders for direction and support, and would generally discern the negative characteristics of narcissistic leaders as less threatening to them (Leary and Baumeister, 2000) and thus as less abusive. For example, a follower low on self-esteem might see a leader taking all the credit for the follower’s success as unfair and abusive, while someone high on self-esteem might interpret this as a signal that they did well and expect that the leader will eventually reciprocate and thus might not always interpret this behavior as being abusive.

Secondly, because of their insecurities about their abilities, low self-esteem individuals, also dubbed as ‘lost souls’ (West and Sweeting, 1997), look toward their leaders for approval and validation and they especially seek charismatic high-power individuals who can help them increase their own self-esteem and offer them direction and clarity (Hayes, 2014; Padilla et al., 2007; Thoroughgood et al., 2012). Narcissistic leaders who tend to exude visionary charisma and come across as confident and dominant (Galvin et al., 2010) would nicely fit that template. This stronger dependence on their (narcissistic) leaders, however, also makes low self-esteem followers more vulnerable to actually *becoming victims of* abusive behavior. Prior research indeed shows that individuals with low self-esteem are less able to defend themselves against aggression (Matthiesen and Einarsen, 2001) and are more likely to become targets of workplace bullying (e.g., Harvey and Keashly, 2003; Bowling and Beehr, 2006; Aquino and Thau, 2009). They are also more likely to avoid confrontation and to conform to social norms (Leary and Baumeister, 2000), have poorer conflict resolution skills (Zapf, 1999), and are susceptible to manipulation especially from authoritarian figures (Gudjonsson and Sigurdsson, 2003; Aquino and Thau, 2009).

Additionally, individuals with low self-esteem might even accept derogatory or aggressive behavior toward them because of their own low perceptions of their self-worth (Padilla et al., 2007; Thoroughgood et al., 2012) and because negative feedback is more consistent with their cognitive structures and expectations (Shrauger, 1975). For instance, low self-esteem individuals are less likely to retaliate against abusive leaders (Tepper, 2007) than those with high self-esteem. Thus, low self-esteem followers’ high need for approval, their tendency to conform to social norms, their dependence on their leader for clarity, direction and validation, their reticence to challenge authority figures and their low self-worth all make followers with low self-esteem “easy targets” for narcissistic leaders’ abuse. Abusive leadership often entails displaced aggression especially toward “safe” targets who are unwilling or unable to defend themselves (Tepper, 2007). Given narcissists’ proclivity to aggress against innocent others when provoked (Martinez et al., 2008), their tendencies for proactive aggression, which constitutes an instrumental use of aggression to exploit others for personal gain (Lobbestael et al., 2014), and their preference for confident others over less confident individuals (e.g., Burton et al., 2017), narcissistic leaders would be more likely to show negative of hostile behavior toward followers with low rather than high self-esteem. Consequently, since followers with low self-esteem are more likely to be affected by narcissistic leaders’ negative characteristics, and also more likely to become chosen as targets of abuse by narcissistic leaders, we expect the following:

*Hypothesis 1:* Follower self-esteem moderates the relationship between leader narcissism and perceived abusive supervision, such that leader narcissism will be positively associated with perceived abusive supervision for followers with low self-esteem, but not for followers with high self-esteem.

We will test this hypothesis in Study 1, a multi-source empirical field study. In Study 2, we will test the same hypothesis using the broader construct of followers’ core self-evaluations, while also examining the consequences of abusive supervision for followers. We will return to this after discussing Study 1 and its results in detail.

The research presented here will make several contributions. Firstly, in focusing on the role of follower personality (i.e., follower self-esteem) in followers’ perceptions of narcissistic leaders, it proposes an important moderator to reconcile previously inconsistent findings. Secondly, this research focuses on *which* followers are especially vulnerable to suffer from the toxic side of narcissistic leaders and who are thus most likely to perceive these leaders as abusive. We thereby further extend the literature on susceptible followers and destructive leaders in general (Padilla et al., 2007; Thoroughgood et al., 2012).

## Method – Study 1

### Sample and Procedure

We used a multi-source field study to test the proposed research model. The sample consisted of 128 followers matched with 85 leaders who worked in different organizations and across different industries (e.g., hospitality, healthcare, and business). Leaders were first approached through Business School graduate student contacts. If they agreed to participate they were then sent a survey link to complete the survey online. The leaders were asked to nominate up to three followers and to provide their email addresses, after which the followers were then forwarded a separate survey link. Surveys could be completed either in English (74% of respondents) or in Dutch (26% of respondents).

The voluntary nature of participation and confidentially was stressed in the accompanying letter for each respondent. The study was carried out in accordance with the recommendations of the Ethics Review Board of the Faculty of Social and Behavioural Sciences of the University of Amsterdam, who approved the protocol for the study. All subjects gave written informed consent in accordance with the Declaration of Helsinki.

The questionnaires were completed anonymously. A unique code was used to match the surveys. To increase the response rate, participants were sent several reminders and leader-follower pairs were offered a small incentive — three pairs would be randomly selected to win a voucher worth 40 euros. Out of 128 leaders who were sent the survey links, 97 completed the survey (response rate 75.8%). In total leaders nominated 203 followers, out of which 128 completed the survey (response rate 63.1%). Leaders (*M*_age_ = 38.38 years, *SD* = 11.09; 69.4% men) had an average tenure of 5.53 (*SD* = 1.90) years and had 34.38 (*SD* = 74.60) followers on average. There were on average 1.51 followers per leader in this sample (observed range 1–3). Followers (*M*_age_ = 35.48 years, *SD* = 12.28; 39.8% men) had an average tenure of 5.79 (*SD* = 7.70) years and had worked with their leader for 2.53 (*SD* = 2.83) years.

### Measures

Leaders filled in the Narcissism personality inventory. Followers filled in the self-esteem personality questionnaire and rated the abusive supervision of the leader.

#### Leader Narcissism

Leaders filled in the 16-item version of the Narcissistic Personality Inventory (NPI-16; Ames et al., 2006). This measure is based on the original 40-item NPI (Raskin and Hall, 1979, 1981), was shown to be psychometrically sound (Ames et al., 2006) and is frequently used to measure narcissism in normal populations (e.g., Peterson et al., 2012; Owens et al., 2015). The scale has a forced choice format, with example items including “I think I am a special person” (narcissistic option = 1) vs. “I am no better or worse than most people” (non-narcissistic option = 0). Especially when items are dichotomous, coefficient alpha can underestimate the reliability of the scale (Raykov et al., 2010). Following recommendation by Widaman et al. (2011) and in line with prior research using NPI-16 with the forced choice variant (e.g., Orth and Luciano, 2015; Orth et al., 2016), we therefore calculated coefficient omega (McDonald, 1999). Coefficient omega of the scale was 0.64. Removing two items increased the reliability coefficient to 0.67 and we used the remaining 14 items in the analyses. The NPI score was computed as the sum of the items, with a higher score indicating higher narcissism.

#### Follower Self-Esteem

Follower self-esteem was measured using the 10-item Rosenberg Scale (Rosenberg, 1965). Example items include: “I feel that I have a number of good qualities” and “I certainly feel useless at times” (reverse item). Responses were given on a four-point Likert scale ranging from 1 (strongly disagree) to 4 (strongly agree). Coefficient alpha of the scale was 0.83.

#### Abusive Supervision

Abusive supervision was measured using the 5-item shortened version (Mitchell and Ambrose, 2007) of Tepper’s (2000) Abusive Supervision measure. Followers indicated their agreement with each item. Examples of items include: “My supervisor ridicules me” and “My supervisor tells me my thoughts and feelings are stupid.” Items were rated on a seven-point Likert scale ranging from 1 (strongly disagree) to 7 (strongly agree). Coefficient alpha of the scale was 0.91.

#### Control Variables

It included followers’ tenure with the leader and gender of the leader and the follower. The negative effects of narcissism may increase over time (Paulhus, 1998), men score higher on narcissism than women (Grijalva et al., 2015b) and followers’ gender is found to be related to perceived victimization, with females reporting more abuse (Aquino and Bradfield, 2000).

### Results – Study 1

#### Confirmatory Factor Analysis

We conducted confirmatory factor analyses to determine whether the data conformed to the assumption that each of the proposed latent variables represents a separate construct. Fitting a measurement model with a large number of indicators (and items) can adversely affect model fit (Hall et al., 1999; Judge et al., 2002). To control for inflated measurement errors caused by multiple items for the latent variable, we divided the items for the personality constructs self-esteem (10) and narcissism (16) into parcels of 3 to 4 items to serve as indicators of the factors using random heterogeneous assignment (Little et al., 2002; Cole et al., 2016). This led to a total of three parcels for CSEs and four parcels for narcissism. The individual scale items were used as indicators of the abusive supervision construct (five items). In addition to the Chi-square statistic, we investigated the Root Mean Square Error of Approximation (RMSEA; acceptable fit: 0.05–0.08, good fit: 0–0.05), the Standardized Root Mean Square Residual (SRMR; acceptable fit: 0.05–0.10, good fit: 0–0.05) and the Comparative Fit Index (CFI; acceptable fit: 0.90–97, good fit: 0.97–1) (see Bentler, 1990; Hu and Bentler, 1999; Schermelleh-Engel et al., 2003; Marsh et al., 2004; Chen et al., 2008).

The CFA supported the proposed 3-factor measurement model, [χ^2^(51, *N* = 128) = 72.11, *p* = 0.03; RMSEA = 0.05; SRMR = 0.07; CFI = 0.96]. Two of the possible alternative models, one in which the items of self-esteem and abusive supervision were merged into an overall factor, and one in which all items loaded on 1 factor, did not converge. A final alternative model, in which the items of follower self-esteem and leader narcissism were merged into an overall factor [χ^2^(53, *N* = 128) = 104.52, *p* < 0.001, RMSEA = 0.07; SRMR = 0.10; CFI = 0.90; Δχ^2^(2) = 32.41, *p* < 0.001], exhibited significantly poorer fit.

#### Hypothesis Testing

**Table 1** presents means, standard deviations and bivariate correlations of the variables. Given the hierarchical structure of our data, with followers (level 1) nested in leaders (level 2), we tested our hypotheses using a random coefficient model. Leader narcissism and follower self-esteem were grand-mean centered. The total variance explained by the models was calculated using the conditional *R*^2^ (Snijders and Bosker, 1994; Nakagawa and Schielzeth, 2013).

**Table 1:** Means, standard deviations, correlations (Study 1).

	*M*	*SD*	1	2	3
**Leader level**					
(1) Leader gender	1.31	0.46			
(2) Leader narcissism	5.49	2.75	-0.10		
**Follower level**					
(1) Follower gender	1.60	0.49			
(2) Tenure with leader	2.53	2.83	-0.12		
(3) Follower self-esteem	3.32	0.41	-0.08	-0.05	
(4) Abusive supervision	1.43	0.75	-0.08	-0.04	-0.25^∗∗^

*N = 128 followers (level 1 data) matched with N = 85 leaders (level 2 data). Tenure in years. Men are coded as 1, women are coded as 2. ^∗^p < 0.05, ^∗∗^p < 0.01.*

**FIGURE 1:**
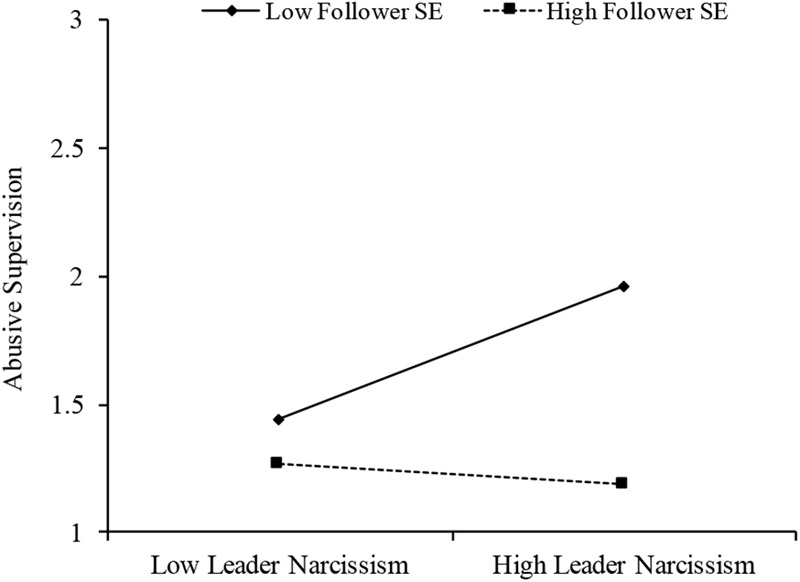
Effects of leader narcissism and follower self-esteem (SE) on abusive supervision (Study 1).

To test the hypothesis, the control variables, leader narcissism, follower self-esteem and their interaction were entered into the random coefficient model. The results of this analysis are presented in **Table 2**. The results showed no main effect of leader narcissism on abusive supervision (*B* = 0.04, *t*(81.06) = 1.54, *p* = 0.127, 95%CI[-0.01, 0.10]), but did show a negative relationship between follower self-esteem and abusive supervision (*B* = -0.57, *t*(120.80) = -3.42, *p* = 0.001, *r* = 0.30, 95%CI[-0.90, -0.24]). As expected, there was a significant interaction found between leader narcissism and follower self-esteem (*B* = -0.14, *t*(112.87) = -2.38, *p* = 0.019, *r* = 0.22, 95%CI[-0.27, -0.02]), which accounted for 3% of the variance in abusive supervision. Subsequent analyses of simple slopes (Aiken and West, 1991) showed that for followers with low self-esteem (1 SD below the mean) the relationship between leader’s narcissism and abusive leadership was positive (*B* = 0.10, *t*(116.84) = 2.71, *p* = 0.008, *r* = 0.24, 95%CI[0.03, 0.17]). For followers with high self-esteem (1 SD above the mean) this relationship was not significant (*B* = -0.02, *t*(108.30) = -0.51, *p* = 0.608, 95%CI[-0.09, 0.05]). See **Figure 1**. Thus, Hypothesis 1 received support, followers with low self-esteem perceived narcissistic leaders as more abusive, those with high self-esteem did not.

**Table 2:** Estimated coefficients of the moderated model (Study 1).

Predictor	*B*	*SE*	*Conditional R^2^*	*B*	*SE*	*Conditional R^2^*
	Abusive supervision (Model 1)			Abusive supervision (Model 2)		
Constant	1.42	0.14		1.43	0.14	
Controls						
Leader gender	0.11	0.16		0.06	0.17	
Follower gender	-0.18	0.13		-0.21	0.13	
Tenure with leader	0.00	0.02		-0.00	0.02	
Predictors						
Leader narcissism	0.04	0.03		0.04	0.03	
Follower self-esteem	-0.57^∗∗^	0.17	0.09^∗∗^	-0.62^∗∗^	0.16	
Interaction						
Leader narcissism × Follower self-esteem				-0.14^∗^	0.06	0.12^∗∗^

*N = 128 followers (level 1 data) matched with N = 85 leaders (level 2 data). ^∗^p < 0.05, ^∗∗^p < 0.01.*

## Study 2

In a second multi-source study we aim to provide a conceptual replication of Study 1 and test whether the stronger relationship of leader narcissism with abusive supervision also occurs for followers who are low on the higher order self-esteem related construct of core self-evaluations (CSEs). In this way, we aim to not only show the robustness of our findings in Study 1, but also to broaden the scope of the research to include a more comprehensive conceptualization of who the potentially most vulnerable followers are (e.g., Padilla et al., 2007; Thoroughgood et al., 2012). In addition, we test the relationship of perceived abusive supervision with followers’ outcomes in order to examine whether vulnerable followers also suffer more negative consequences under narcissistic leaders.

### Leader Narcissism and Follower Core Self-Evaluations

Core self-evaluations is a more general higher order construct which, in addition to self-esteem, comprises of self-efficacy, locus of control and emotional stability and refers to “basic conclusions or bottom-line evaluations that individuals hold about themselves” (Judge and Bono, 2001, p. 81). Individuals with more positive CSEs like themselves and think of themselves as capable, worthy, and competent in dealing with issues in different contexts (Judge et al., 2003). Conversely, individuals with more negative CSEs dislike themselves and are not confident in their capabilities, competence, or worthiness. Having lower or more negative self-evaluations, similarly as with followers with low self-esteem, makes such followers more susceptible to suffer from abusive or destructive leaders (Luthans et al., 1998; Padilla et al., 2007; Thoroughgood et al., 2012). For instance, having low expectations of one’s ability to perform well (i.e., low self-efficacy) increases the followers’ dependence on their leaders because these individuals are more likely to feel they need the leaders to provide them with clarity and direction (Thoroughgood et al., 2012). Similarly, having a belief that outcomes are the result of external events (i.e., external locus of control) instead of one’s own actions makes individuals easier to manipulate, and also makes them more likely to seek out powerful others who can take care of them and to whom they can defer responsibility (Padilla et al., 2007). Thus, we expect that we can extend the construct of vulnerable followers from low self-esteem to include those individuals who have more general negative views regarding not only their self-worth, but also their competencies and feelings of control over outcomes (i.e., those followers with low CSEs). This leads us to the following hypothesis:

*Hypothesis 2:* Follower CSE moderates the relationship between leader narcissism and abusive supervision, such that leader narcissism will be positively associated with perceived abusive supervision for followers with low CSEs, but not for followers with high CSEs.

### Consequences of Abusive Supervision

Abusive supervision has been shown to have many detrimental consequences for followers, such as psychological distress (e.g., strain, emotional exhaustion, and depression), lower family well-being, and higher turnover intentions (Tepper, 2000, 2007; Aryee et al., 2008; Wu and Hu, 2009; Carlson et al., 2012; Schyns and Schilling, 2013). Abusive supervision has also been linked to lower follower job performance, both with respect to reduced core task performance as well as reduced organizational citizenship behavior (Zellars et al., 2002; Aryee et al., 2007; Harris et al., 2007; Tepper, 2007; Xu et al., 2012; Schyns and Schilling, 2013).

In Study 2 we include the consequences of abusive supervision and test whether perceptions of abusive supervision relate to distress and job performance. Specifically, we focus on followers’ self-rated emotional exhaustion, as being reflective of their experienced psychological distress, as well as their task performance as rated by their leaders. Given the argumentation presented above we expect that leader narcissism, through greater perceived abusive supervision, will be associated with greater emotional exhaustion and worse performance, especially for followers with low CSEs. Combining the arguments presented above in the development of Hypothesis 2 we thus propose a moderated mediation model and argue that leader narcissism has an indirect negative effect on follower performance and emotional exhaustion, via perceptions of abusive supervision, and that this indirect effect is contingent on followers’ CSEs.

*Hypothesis 3:* Leader narcissism is related to follower task performance via a conditional indirect effect, such that the negative indirect effect via abusive supervision on performance is stronger when follower CSE is low rather than high.
*Hypothesis 4:* Leader narcissism is related to follower exhaustion via a conditional indirect effect, such that the positive indirect effect on exhaustion via abusive supervision is stronger when follower CSE is low rather than high.

**FIGURE 2:**
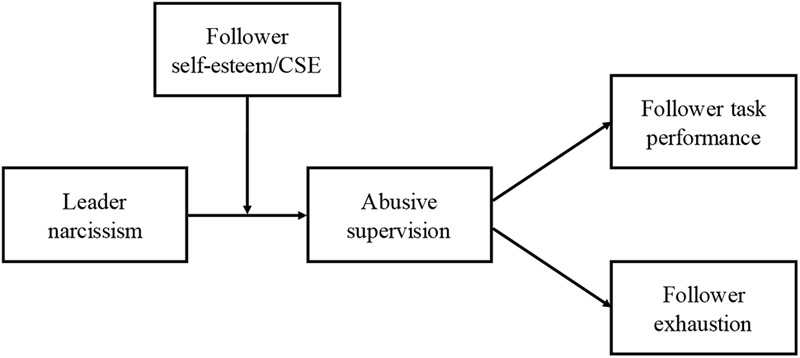
Proposed moderated mediation model (CSE, core self-evaluation).

To sum up, we propose, in replication of the findings of Study 1 that because of their greater reliance on external cues and dependence on narcissistic leaders, followers with low general CSEs will be more likely to perceive narcissistic leaders as abusive. Furthermore, as a consequence, low CSE followers are more likely to suffer negative outcomes in terms of psychological distress as well as lower performance as a result of leader narcissism. **Figure 2** presents the full proposed model.

### Method – Study 2

#### Sample and Procedure

We performed a multi-source field study to test the proposed moderated mediation research model. The sample consisted of 176 unique leader-follower dyads working in a wide range of jobs (lawyers, salespersons, account managers) in different organizations (e.g., health care, government, insurance) in the Netherlands. These contacts were approached through Business School graduate student contacts. Survey packets were sent to both the supervisor and the employee and the voluntary nature of participation and confidentially was stressed in the accompanying letter for each respondent. The study was carried out in accordance with the recommendations of the Economics and Business Ethics Committee, University of Amsterdam, who approved the protocol for the study. All subjects gave written informed consent in accordance with the Declaration of Helsinki.

The questionnaires were completed anonymously. Individual surveys could be returned directly to the researchers and a unique code was used to match the surveys. In total, 179 of the contacted supervisors and 186 of the employees returned fully filled out questionnaires, resulting in a response rate of 69% for complete dyads. Most leaders (Mean age 42.35 years, Mean tenure 9.00 years) were male (58.5%), and most followers (Mean age 33.84 years, Mean tenure 5.79 years) were female (56.3%).

#### Measures

Unless otherwise indicated, all items were rated on a seven-point Likert scale ranging from 1 (strongly disagree) to 7 (strongly agree). Leaders filled in the Narcissism personality inventory and rated followers’ task performance. Followers filled in the CSE personality questionnaire, rated the abusive supervision of the leader and indicated their feelings of exhaustion.

##### Leader narcissism

Similarly as in Study 1, leaders filled in the 16-item version of the Narcissistic Personality Inventory (NPI-16; Ames et al., 2006). Consistent with recent research that suggests that a Likert response format to the NPI results in stronger reliabilities (Miller et al., 2017) and our experiences in Study 1, in Study 2 we replaced the forced-choice response by a seven point Likert format (cf. Penney and Spector, 2002; Moon et al., 2016), with 1 = strongly disagree through 7 = strongly agree. High NPI scores indicate higher levels of narcissism. A sample item of a narcissistic response is “I am apt to show off if I get a chance.” Coefficient alpha of the scale was 0.88.

##### Follower performance

Leaders also provided ratings for the focal follower’s performance using four items from Pearce and Porter (1986, see also Ashford and Black, 1996). Leaders were asked to report how the follower was rated relative to others on a percentage basis at their last actual performance evaluation (e.g., 60th percentile, 70th percentile). A sample item is “The achievement of work goals.” Coefficient alpha of the scale was 0.85.

##### Follower core self-evaluations (CSEs)

We measured followers’ CSEs with the 12- item scale developed and validated by Judge et al. (2003). The scale measures positive feelings about the self in terms of self-esteem, generalized self-efficacy, emotional stability, and locus of control. Examples of items are: “Overall, I am satisfied with myself” and “I am capable of coping with most of my problems.” The coefficient alpha in this study was 0.78.

##### Abusive supervision

Abusive supervision was measured using the 5-item shortened version (Mitchell and Ambrose, 2007) of Tepper’s (2000). Abusive Supervision measure. Followers indicated their agreement with each item. Examples of items are: “ridicules me” and “tells me my thoughts and feelings are stupid.” Coefficient alpha of the scale was 0.92.

##### Follower exhaustion

Followers’ emotional exhaustion was assessed with the Dutch version (Schaufeli and van Dierendonck, 2000) of the Exhaustion scale of the Maslach Burnout Inventory–General Survey (Schaufeli et al., 1996). A sample item is “I feel mentally exhausted by my work” Coefficient alpha of the scale was 0.84.

##### Control variables

Control variables were the same as in Study 1, namely followers’ tenure with the leader and gender of the leader and the follower.

### Results – Study 2

#### Confirmatory Factor Analysis

We again conducted confirmatory factor analyses to determine whether the data conformed to the assumption that each of the proposed latent variables represents a separate construct. To control for inflated measurement errors caused by multiple items for the latent variable, we divided the items for the personality constructs CSEs (12) and narcissism (16) into parcels of four items to serve as indicators of the factors using random heterogeneous assignment (Little et al., 2002; Cole et al., 2016). This lead to a total of three parcels for CSEs and four parcels for narcissism. The individual scale items were used as indicators of the abusive supervision (five items), performance (four items), and exhaustion factors (five items).

The CFA supported the proposed 5-factor measurement model, [χ^2^(179, *N* = 176) = 349.39, *p* < 0.001; RMSEA = 0.07; SRMR = 0.07; CFI = 0.92]. Two alternative models, one in which the items of follower performance and exhaustion were merged into an overall factor (χ^2^(183, *N* = 176) = 714.79, *p* < 0.001, RMSEA = 0.13; SRMR = 0.16; CFI = 0.76; Δχ^2^(4) = 365.4, *p* < 0.001], and one in which the items of follower exhaustion and abusive supervision were merged into an overall factor [χ^2^(183, *N* = 176) = 656.88, *p* < 0.001, RMSEA = 0.12; SRMR = 0.132; CFI = 0.79; Δχ^2^(4) = 307.49, *p* < 0.001] exhibited significantly poorer fit. We also compared the proposed 5-factor measurement model with a two-factor model, which had the items of leader narcissism and follower performance (all rated by the leader) loading on the same factor and the items rated by the follower (CSE, abusive supervision and exhaustion) loading on a separate factor. Again, the 5-factor measurement model showed a significantly better fit over the alternative model [χ^2^(188, *N* = 176) = 1290.89, *p* < 0.001, RMSEA = 0.18; SRMR = 0.18; CFI = 0.51; Δχ^2^(9) = 941.50, *p* < 0.001).

#### Hypothesis Testing

**Table 3** presents means, standard deviations and bivariate correlations of the variables. To test the hypotheses relating to our moderated mediation model, we follow the procedure outlined by Preacher et al. (2007). Specifically, we use the MODMED macro (Model 7, Preacher and Hayes, 2004), which provides results relevant for our hypotheses in three steps. Leader narcissism and follower CSE were centered at the mean for all analyses. Before employing the MODMED macro to test our hypotheses, we ran a regression analysis including the controls and leader narcissism and follower CSE in order to test for main effects (see **Table 4**, Model 1). The results showed no significant main effect of leader narcissism on abusive supervision [*B* = 0.18, *t*(170) = 1.88, *p* = 0.062, 95%CI[-0.01,0.36]), but did show a negative relationship between follower CSEs and abusive supervision (*B* = -0.25, *t*(170) = -2.31, *p* = 0.022, *r* = 0.17, 95%CI[-0.47, -0.04]).

**Table 3:** Means, standard deviations, correlations (Study 2).

	*M*	*SD*	1	2	3	4	5	6	7
(1) Tenure with leader	3.08	3.48							
(2) Leader gender	1.41	0.49	-0.26^∗∗^						
(3) Follower gender	1.56	0.50	0.00	0.28^∗∗^					
(4) Leader narcissism	4.01	0.90	-0.10	-0.16^∗^	-0.14				
(5) Follower CSE	5.12	0.75	0.03	0.06	-0.12	-0.06			
(6) Abusive supervision	1.71	1.12	-0.06	-0.21^∗∗^	-0.07	0.19^∗^	-0.19^∗^		
(7) Follower performance	7.61	1.19	0.08	-0.04	-0.11	-0.05	0.11	-0.27^∗∗^	
(8) Follower exhaustion	2.76	1.18	-0.05	0.02	0.04	0.28^∗∗^	-0.44^∗∗^	0.29^∗∗^	-0.11

*N = 176 dyads. Tenure in years. Men are coded as 1, women are coded as 2. CSE, core self-evaluation. ^∗^p < 0.05, ^∗∗^p < 0.01.*

**Table 4:** Estimated coefficients of main effects and moderation on abusive supervision (Study 2).

Predictor	*B*	*SE*	*F*		*R*^2^
	**Abusive supervision (Model 1)**				
Constant	2.48	0.35			
Controls					
Leader gender	-0.04	0.17			
Follower gender	-0.44^∗^	0.18			
Tenure with leader	-0.03	0.02			
Predictors					
Leader narcissism	0.18	0.09			
Follower CSE	-0.25^∗^	0.11	4.06		0.11^∗∗^

	**Abusive supervision (Model 2)**				
Constant	2.57	0.34			
Controls					
Leader gender	-0.11	0.17			
Follower gender	-0.45^∗^	0.18			
Tenure with leader	-0.03	0.02			
Predictors					
Leader narcissism	0.20^∗^	0.09			
Follower CSE	-0.26^∗^	0.11			
Interaction					
Leader narcissism × Follower CSE	-0.33^∗^	0.13	4.61	0.14^∗∗^	

*N = 176 dyads. CSE, core self-evaluation. ^∗^*p* < 0.05, ^∗∗^*p* < 0.01.*

**FIGURE 3:**
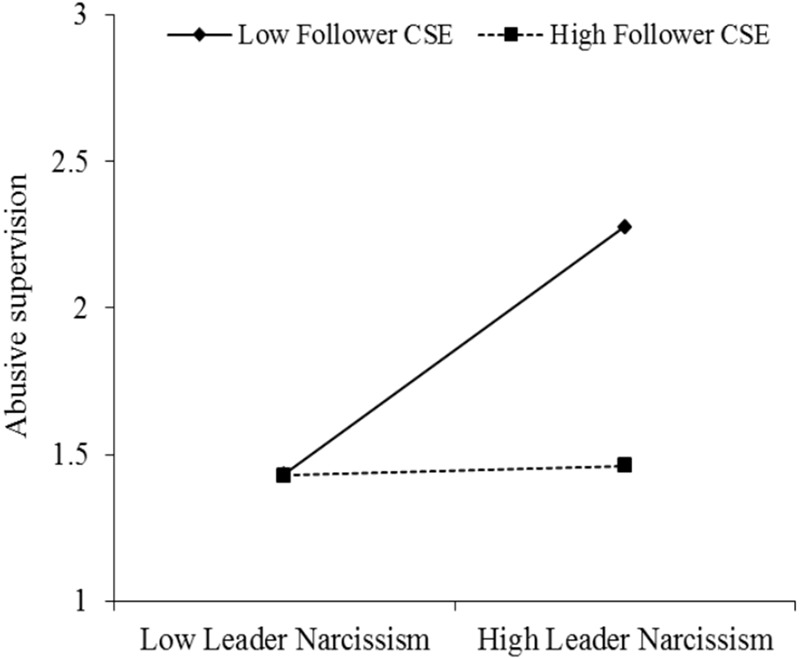
Effects of leader narcissism and follower CSE on abusive supervision (Study 2).

To test Hypothesis 2, the first step of the MODMED analysis examines the effect of the interaction between leader narcissism and follower CSEs on abusive supervision. Results are presented in **Table 4** (Model 2) and reveal a significant interaction between leader narcissism and CSEs of the follower (*B* = -0.33, *t*(169) = -2.59, *p* = 0.011, *r* = 0.20, 95%CI[-0.58, -0.08]) that accounts for 3% of the variance in abusive supervision. We assessed the nature of this significant interaction by plotting values representing plus and minus 1 standard deviation from the means for leader narcissism and follower CSEs. As shown in **Figure 3** and supported by a simple slopes test (Aiken and West, 1991), leader narcissism was positively related to abusive supervision when follower CSEs are low (1 SD below the mean, *B* = 0.45, *t*(169) = 3.21, *p* = 0.002, *r* = 0.24, 95%CI[0.17, 0.72]) and this relationship weakened and became non-significant for followers high on CSEs (1 SD above the mean, *B* = -0.05, *t*(169) = -0.37, *p* = 0.713, 95%CI[-0.29, 0.20]), supporting Hypothesis 2.

To test the moderated mediation model as formalized in Hypothesis 3 and 4, the second step of the MODMED procedure (**Table 5**) examines the impact of abusive supervision on follower task performance and exhaustion, while controlling for leader narcissism. As expected, abusive supervision was negatively related to follower task performance (*B* = -0.30, *t*(170) = -3.72, *p* < 0.001, *r* = 0.27, 95%CI[-0.46, -0.14]) and positively related to exhaustion (*B* = 0.28, *t*(170) = 3.58, *p* < 0.001, *r* = 0.26, 95%CI[0.13, 0.43]). The third step of the MODMED procedure examines the significance of the conditional indirect effect of leader narcissism on task performance and exhaustion through abusive supervision as a function of follower CSEs. The proposed model receives support if the conditional indirect effect of leader narcissism on task performance and exhaustion, via abusive supervision differs in strength across low and high levels of follower CSEs. We indeed found such support as the index of moderated mediation is significant (Hayes, 2014), meaning that the indirect relationship of leader narcissism with task performance and exhaustion through abusive supervision was found to be a function of follower CSEs (performance: Index = 0.10; Bias and accelerated 95% CI[0.02, 0.20]; exhaustion: Index = -0.09; Bias and accelerated 95% CI[-0.22, -0.01]). Specifically, there was a negative effect of leader narcissism on follower task performance (*B* = -0.13; Bias and accelerated 95% CI[-0.28, -0.03]) and a positive effect on follower exhaustion (*B* = 0.12; Bias and accelerated 95% CI[0.03, 0.28]) via abusive supervision when follower CSEs were low, and no significant effect of leader narcissism on follower task performance (*B* = 0.01; Bias and accelerated 95% CI[-0.06, 0.07]) and exhaustion (*B* = -0.01; Bias and accelerated 95% CI[-0.09, 0.04]) via abusive supervision when follower CSEs were high (see also **Table 6**).

**Table 5:** Estimated coefficients of mediation (Study 2).

Predictor	*B*	*SE*	*F*		*R*^2^
	**Follower task performance**				
Constant	8.69	0.42			
Controls					
Leader gender	-0.27	0.18			
Follower gender	-0.13	0.20			
Tenure with leader	0.02	0.03			
Predictors					
Abusive supervision	-0.30^∗∗^	0.08			
Leader narcissism	-0.02	0.10	3.56		0.09^∗∗^

	**Follower exhaustion**				
Constant	1.66	0.41			
Controls					
Leader gender	0.16	0.18			
Follower gender	0.25	0.19			
Tenure with leader	0.01	0.03			
Predictors					
Abusive supervision	0.28^∗∗^	0.08			
Leader narcissism	0.34^∗∗^	0.10	6.01	0.15^∗∗^	

*N = 176 dyads. ^∗^p < 0.05, ^∗∗^p < 0.01.*

**Table 6:** Bootstrapping results for test of conditional indirect effects on follower task performance and exhaustion at specific values of the moderator (CSE): Mean and ±+1 standard deviation (Study 2).

Follower task performance				95% CI	
Mediator	Value of CSE	Conditional indirect effect	*SE*	Lower	Upper
Abusive supervision	-1 *SD* (-0.75)	-0.13^∗^	0.06	-0.28	-0.03
	*M* (0.00)	-0.06	0.04	-0.15	0.00
	+1 *SD* (0.75)	0.01	0.03	-0.06	0.07

**Follower exhaustion**				**95% CI**	
**Mediator**	**Value of CSE**	**Conditional indirect effect**	***SE***	**Lower**	**Upper**

Abusive supervision	-1 *SD* (-0.75)	0.12^∗^	0.06	0.03	0.28
	*M* (0.00)	0.06^∗^	0.03	0.01	0.14
	+1 *SD* (0.75)	-0.01	0.03	-0.09	0.04

*Results are based on 5000 bootstrap samples. Conditional indirect effects are two-tailed. CI, confidence interval; CSE, core self-evaluation. ^∗^p < 0.05.*

Thus, as predicted, when follower CSEs are low, leader narcissism is positively related to perceived abusive supervision, and abusive supervision in turn is negatively related to follower task performance and positively to follower exhaustion. When follower CSEs are high, the positive relationship with abusive supervision becomes insignificant and there is no longer an indirect effect through abusive supervision on task performance and exhaustion for leader narcissism.

## Discussion

By focusing on follower self-esteem and follower CSEs, we sought to reconcile the inconsistent findings regarding followers’ perceptions of narcissistic leaders and at the same time identify followers who are more or less vulnerable to narcissistic leaders. Despite the fact that narcissistic leaders have many negative characteristics that may predispose them to being abusive toward their followers (e.g., lack of empathy, sense of entitlement, exploitativeness, and aggressive tendencies), using two multi-source field studies we consistently found that narcissistic leaders were *only* perceived as abusive by followers with low self-esteem (Study 1), and followers who were lower on the higher order construct of CSEs (Study 2). Moreover, when these vulnerable followers perceived more abusive leader behavior when working under leaders high on the trait of narcissism, they also showed poorer functioning at work. They reported having higher psychological distress, as reflected in their greater emotional exhaustion, and they were rated by their leaders as having lower task performance (Study 2). Followers with high self-esteem or high CSEs, seemed to be less negatively affected by narcissistic leaders. They did not perceive narcissistic leaders as more abusive, nor did they, as a result of this, show worse functioning at work.

Our research extends prior work in several ways. Firstly, we show that follower personality plays a critical role in determining how followers perceive and experience narcissistic leaders. This provides one explanation as to why prior research has tended to find inconsistencies when looking at followers’ evaluations of their narcissistic leaders, with followers sometimes perceiving narcissistic leaders positively or neutrally (Judge et al., 2006; Nevicka et al., 2011b; Owens et al., 2015) and sometimes negatively (Judge et al., 2006; Martin et al., 2016). Additionally, we contribute to literature on leader narcissism which has sought to ascertain what kind of impact narcissistic leaders have on those that they lead (Campbell and Campbell, 2009; Judge et al., 2009; Sedikides and Campbell, 2017), particularly given that they have a paradoxical mixture of positive and negative characteristics. By examining followers’ perceptions of abusive supervision, we show that whether or not narcissistic leaders affect their followers negatively depends at least in part on followers’ personality traits. In focusing on followers’ self-esteem and their CSEs we show that people’s fundamental appraisals regarding their own self-worth, competence, capabilities and the extent to which they feel in control of their lives (Chang et al., 2012), influence whether they are affected by the toxic side of narcissistic leaders. Those low on self-esteem and CSEs seem to be more vulnerable likely in both needing more direction, while also perceiving narcissistic leaders as more threatening. Our findings also suggest that individuals with higher self-esteem and high CSEs are better able to cope with the toxic side of narcissistic leaders and perceive them as less threatening, than those low in self-esteem and CSEs. As such, a person’s positive self-appraisals may provide them with a buffer in dealing with narcissists’ negative side.

Secondly, our findings further inform research on susceptible followers and the initiation and persistence of destructive leadership styles in organizations (Padilla et al., 2007; Thoroughgood et al., 2012). We show that certain personality traits make followers particularly vulnerable to perceiving and/or encountering leader abuse when working with destructive leaders such as narcissistic leaders. We not only demonstrate that followers with low self-esteem and more negative CSEs perceive more abusive behavior when working under narcissistic leaders, but also that as a result of this, narcissistic leaders have significant negative ramifications on such followers’ daily functioning at work, both in terms of their psychological distress as well as their work performance.

Finally, our research can help inform literature on abusive supervision and workplace victimization in general (Tepper, 2007; Martinko et al., 2013) by identifying how dispositional leader-level and follower-level characteristics interact to influence followers’ experience of abusive supervisory behavior. For instance, prior research on abusive supervision found that leaders with lower emotional intelligence (Xiaqi et al., 2012), as well as higher Machiavellianism and higher psychopathy (Kiazad et al., 2010; Wisse and Sleebos, 2016) were perceived as more abusive. The results of our studies show that narcissism is an important addition to the list of characteristics which may make leaders more predisposed toward abusive behaviors, however, in the case of narcissistic leaders this only holds *provided that* these leaders are coupled with followers who see themselves as low in self-worth and competence. Thus, our findings suggest that the negative impact of narcissistic leaders is only manifested when there are vulnerable ‘targets’ available.

### Strengths, Limitations, and Future Research

The main strength of our research lies in the replication of findings across two heterogeneous samples as well as an extension of our moderator from self-esteem to the more general higher order construct of CSEs. This consistent pattern of findings is noteworthy given the acknowledged difficulty in detecting moderation within field settings (McClelland and Judd, 1993). Furthermore, given that the samples were drawn from diverse workplace settings and industries, this lends strength to the generalizability and robustness of the results.

Another methodological strength was the use of multi-source measurement which reduces concerns regarding common source variance with respect to inflating the main effects found between predictors and the dependent variables as well as regarding the attenuation of the interaction effects (Siemsen et al., 2010).

Although our two studies show consistent results and enhance our understanding regarding the interplay of leader and follower dispositional characteristics on perceived abusive leadership by followers, they are not without limitations. Firstly, while our theory provides a strong indication as to the direction of the proposed relationships, the cross-sectional nature of our data prevents assertions of causality. For example, an alternative explanation to our findings in Study 2 could be that followers with low self-esteem and low CSEs receive abusive supervision from narcissistic leaders *because* they are perceived to be performing less well than followers with high self-esteem and high CSEs. Narcissists are overly critical of others and demand perfectionism (Stoeber et al., 2015), thus, insofar as narcissistic leaders feel that the performance of their followers is reflective of their own success, they may indeed wish to punish low performing followers. Future studies could employ a longitudinal design and measure follower job performance over time to examine how lower or higher follower job performance ratings or evaluations subsequently influence different followers’ perceptions of abuse from narcissistic leaders.

Secondly, because abusive supervision as it was measured in our studies concerns followers’ subjective perceptions regarding a leader’s mistreatment, it may not reflect the actual levels of mistreatment. Thus, narcissistic leaders could be actually behaving more abusively toward those with low self-esteem and low CSEs, or these followers may simply be more attuned to potential victimization (Aquino and Thau, 2009) and as such experience narcissists’ dominance, lack of empathy and egocentrism as abusive. Nonetheless, researchers have argued that follower perceptions are critical to include in measures of abusive supervision because leader behavior can only have an effect on followers if it is also perceived by them (Schyns and Schilling, 2013). Future research could further disentangle perceptions of abuse and actual abuse by asking leaders to report on their abusive behavior in relation to specific followers. Another possibility would be to use an experimental paradigm in which actual abusive behavior is observed and contrasted with perceptions of abusive behavior. Given our argumentation that narcissistic leaders would perceive followers with low self-esteem and low CSEs as easy targets, we would expect leader narcissism to be positively related to more actual reported abusive behavior toward these vulnerable followers.

Thirdly, in order to obtain multiple followers, in Study 1 the leaders were asked to nominate followers who would fill out the questionnaire. This might have inadvertently led to a selection bias as leaders might have chosen only those followers with whom they had a good relationship. However, given the consistent findings across both of our studies, it does not appear that this potential bias overrode or influenced the found results.

Finally, because the focus of our research was solely on narcissistic leaders, we did not measure the other two Dark Triad traits (Machiavellianism and psychopathy; Paulhus and Williams, 2002). Therefore, it was not possible to control for these constructs to isolate the unique effects of leader narcissism. Future research should consider measuring all three of the dark triad traits simultaneously to examine whether our findings generalize uniformly or differentially to the other two dark triad traits.

Our research focused on the impact of leader narcissism on followers’ emotional exhaustion and task performance (Study 2). Future studies could test our model using other important outcome variables that are known to be affected by abusive supervision, such as followers’ job satisfaction, organizational commitment, job stress, vitality, turnover intentions and organizational citizenship behavior (Tepper, 2007; Martinko et al., 2013; Schyns and Schilling, 2013; Mackey et al., 2015). Prior research has shown that due to the sense of injustice that they feel, followers tend to retaliate in response to abusive supervision in the form of deviant behavior such as aggression, theft and sabotage (e.g., Tepper et al., 2008, 2009; Liu et al., 2010; Burton and Hoobler, 2011). Thus, another interesting avenue of research would be to examine whether or not vulnerable followers (i.e., those with low self-esteem and low CSEs) would show such retaliation toward narcissistic leaders. Because individuals with low self-esteem are in general reluctant to engage in confrontation (Gudjonsson and Sigurdsson, 2003) particularly with authority figures, and because their feelings of low self-worth may lead them to believe that abusive behavior is justified (Padilla et al., 2007; Thoroughgood et al., 2012), such vulnerable individuals might be less likely to retaliate against narcissistic leaders, at least in an overt manner. Moreover, prior research shows that it is individuals with high (unstable) self-esteem who are more likely to react aggressively to threats to their ego rather than those with low self-esteem (Bushman and Baumeister, 1998; Bushman et al., 2009). The reason is that low self-esteem individuals tend to be more cautious and risk-averse in their responses, which makes them unlikely to react aggressively (Baumeister et al., 2000).

A final fruitful direction for future research would be to more closely examine the formation of dependence between susceptible followers and destructive leaders, such as narcissistic leaders. Because of their strong need for affirmation, desire for clarity, direction and higher self-esteem, the so-called ‘lost souls’ seek out charismatic and powerful leaders and thereby make themselves vulnerable to abuse by such leaders (Hayes, 2014; Padilla et al., 2007; Thoroughgood et al., 2012). Their high psychological need for such leaders also makes it likely that they will become dependent on such leaders. We know for instance that followers’ personal identification with their transformational leaders (i.e., extent to which an individual’s belief about the leader is self-referential) fosters greater dependence on those leaders rather than empowerment (Kark et al., 2003). It would be interesting to examine whether followers with low self-esteem and negative CSEs show greater dependence on (narcissistic) leaders, and what effects this has, for example in terms of stifling employee voice.

### Practical Implications and Conclusion

This research has several practical implications for organizations. First, given the negative impact of narcissistic leaders on vulnerable followers, organizations could consider obtaining narcissism ratings of job applicants and restricting narcissists’ entry to leadership functions, or getting rid of narcissistic leaders altogether. In light of the current findings, avoiding narcissistic individuals in leadership positions might appear to be an attractive alternative, however, narcissistic individuals also have positive characteristics which could make them useful for organizations in certain contexts (Sedikides and Campbell, 2017). For example, narcissists promote bold visions and are charismatic, they tend to persist in the face of failure, and they are good in crisis management (Galvin et al., 2010; Watts et al., 2013). Thus, a more fruitful alternative might be for organizations to attempt to find the best fit between managers and their subordinates. For example, if project teams are being formed then organizations should consider allocating subordinates with lower self-esteem or negative CSEs to project leaders who are lower on narcissism. Additionally, because vulnerable followers are more likely to perceive abusive behavior from narcissistic leaders and might be reluctant to speak out about this, it is important for such employees to be provided with support networks and means of voicing their concerns and feelings. Organizations could, for example, provide these employees with support groups, or a mentor that they could safely talk to when in need. From a preventative perspective, trainings on increasing self-confidence, self-esteem, and self-efficacy could be initiated to help these employees become more resilient to narcissistic leaders.

To conclude, we show that despite having many negative characteristics such as egocentrism, aggression, exploitativeness and lack of empathy, narcissistic leaders do not indiscriminately negatively affect *all* people they lead. In fact, the toxic effects of narcissistic leaders in terms of perceived abusive supervision, seem to be only experienced by vulnerable followers who have low self-esteem or low core self-evaluations. This research thus helps shed light on the consequences of narcissistic leaders for those they lead and identify *which* followers are more or less susceptible to experiencing the dark side of these leaders.

## Author Contributions

BN, ADH, DDH, and FB conceived and developed the project, contributed to the interpretation of the results. Data collection was coordinated and conducted by BN and ADH. BN and ADH performed the data analyses. BN drafted the manuscript. ADH, DDH, and FB provided the critical revisions. All authors agreed to all aspects of the work and approved the final version of the manuscript.

## Conflict of Interest Statement

The authors declare that the research was conducted in the absence of any commercial or financial relationships that could be construed as a potential conflict of interest. The reviewer SS and handling Editor declared their shared affiliation.

## References

[B1] Aiken L. S. West S. G. (1991). *Multiple Regression: Testing and Interpreting Interactions.* Newbury Park, CA: Sage.

[B2] Ames D. R. Rose P. Anderson C. P. (2006). The NPI-16 as a short measure of narcissism. *J. Res. Pers.* 40 440–450. 10.1016/j.jrp.2005.03.002 23815119

[B3] Aquino K. Bradfield M. (2000). Perceived victimization in the workplace: The role of situational factors and victim characteristics. *Organ. Sci.* 11 525–537. 10.1287/orsc.11.5.525.15205

[B4] Aquino K. Thau S. (2009). Workplace victimization: aggression from the target’s perspective. *Annu. Rev. Psychol.* 60 717–741. 10.1146/annurev.psych.60.110707.163703 19035831

[B5] Aryee S. Chen Z. X. Sun L. Y. Debrah Y. A. (2007). Antecedents and outcomes of abusive supervision: test of a trickle-down model. *J. Appl. Psychol.* 92 191–201. 10.1037/0021-9010.92.1.191 17227160

[B6] Aryee S. Sun L. Y. Chen Z. X. G. Debrah Y. A. (2008). Abusive supervision and contextual performance: the mediating role of emotional exhaustion and the moderating role of work unit structure. *Manag. Organ. Rev.* 4 393–411. 10.1111/j.1740-8784.2008.00118.x

[B7] Ashford S. J. Black J. S. (1996). Proactivity during organizational entry: the role of desire for control. *J. Appl. Psychol.* 81 199–214. 10.1037/0021-9010.81.2.199

[B8] Avey J. B. Palanski M. E. Walumbwa F. O. (2011). When leadership goes unnoticed: the moderating role of follower self-esteem on the relationship between ethical leadership and follower behavior. *J. Bus. Ethics* 98 573–582. 10.1007/s10551-010-0610-2

[B9] Back M. D. Schmukle S. C. Egloff B. (2010). Why are narcissists so charming at first sight? Decoding the narcissism–popularity link at zero acquaintance. *J. Pers. Soc. Psychol.* 98 132–145. 10.1037/a0016338 20053038

[B10] Baumeister R. F. Bushman B. J. Campbell W. K. (2000). Self-esteem, narcissism, and aggression: does violence result from low self-esteem or from threatened egotism? *Curr. Dir. Psychol. Sci.* 9 26–29. 10.1111/1467-8721.00053

[B11] Bentler P. M. (1990). Comparative fit indexes in structural models. *Psychol. Bull.* 107 238–246. 10.1037/0033-2909.107.2.238 2320703

[B12] Bowling N. Beehr T. (2006). Workplace harassment from the victim’s perspective: a theoretical model and meta-analysis. *J. Appl. Psychol.* 91 998–1012. 10.1037/0021-9010.91.5.998 16953764

[B13] Brockner J. (1988). *Self-Esteem at Work: Research, Theory, and Practice.* Lexington, MA: Lexington Press.

[B14] Brunell A. B. Gentry W. A. Campbell W. K. Hoffman B. J. Kuhnert K. W. DeMarree K. G. (2008). Leader emergence: the case of the narcissistic leader. *Pers. Soc. Psychol. Bull.* 34 1663–1676. 10.1177/0146167208324101 18794326

[B15] Burton J. P. Hoobler J. M. (2011). Aggressive reactions to abusive supervision: the role of interactional justice and narcissism. *Scand. J. Psychol.* 52 389–398. 10.1111/j.1467-9450.2011.00886.x 21504430

[B16] Burton K. A. Adams J. M. Hart W. Grant B. Richardson K. Tortoriello G. (2017). You remind me of someone awesome: narcissistic tolerance is driven by perceived similarity. *Pers. Individ. Dif.* 104 499–503. 10.1016/j.paid.2016.09.019

[B17] Bushman B. J. Baumeister R. F. (1998). Threatened egotism, narcissism, self-esteem, and direct and displaced aggression: does self-love or self-hate lead to violence? *J. Pers. Soc. Psychol.* 75 219–229. 10.1037/e552692012-026 9686460

[B18] Bushman B. J. Baumeister R. F. Thomaes S. Ryu E. Begeer S. West S. G. (2009). Looking again, and harder, for a link between low self-esteem and aggression. *J. Pers.* 77 427–446. 10.1111/j.1467-6494.2008.00553.x 19192074

[B19] Campbell W. K. Campbell S. M. (2009). On the self-regulatory dynamics created by the peculiar benefits and costs of narcissism: a contextual reinforcement model and examination of leadership. *Self Identity* 8 214–232. 10.1080/15298860802505129

[B20] Carlson D. Ferguson M. Hunter E. Whitten D. (2012). Abusive supervision and work–family conflict: the path through emotional labor and burnout. *Leader. Q.* 23 849–859. 10.1016/j.leaqua.2012.05.003

[B21] Carlson E. N. Vazire S. Oltmanns T. F. (2011). You probably think this paper’s about you: narcissists’ perceptions of their personality and reputation. *J. Pers. Soc. Psychol.* 101 185–201. 10.1037/a0023781 21604895 PMC3119754

[B22] Chang C. H. Ferris D. L. Johnson R. E. Rosen C. C. Tan J. A. (2012). Core self-evaluations: a review and evaluation of the literature. *J. Manag.* 38 81–128. 10.1002/job.761

[B23] Chen F. Curran P. J. Bollen K. A. Kirby J. Paxton P. (2008). An empirical evaluation of the use of fixed cutoff points in RMSEA test statistic in structural equation models. *Sociol. Methods Res.* 36 462–494. 10.1177/0049124108314720 19756246 PMC2743032

[B24] Cole D. A. Perkins C. E. Zelkowitz R. L. (2016). Impact of homogeneous and heterogeneous parceling strategies when latent variables represent multidimensional constructs. *Psychol. Methods* 21 164–174. 10.1037/met0000047 26323000

[B25] Elangovan A. R. Xie J. L. (1999). Effects of perceived power of supervisor on subordinate stress and motivation: the moderating role of subordinate characteristics. *J. Organ. Behav.* 20 359–373. 10.1002/(SICI)1099-1379(199905)20:3<359::AID-JOB902>3.0.CO;2-Z

[B26] Galvin B. M. Waldman D. A. Balthazard P. (2010). Visionary communication qualities as mediators of the relationship between narcissism and attributions of leader charisma. *Pers. Psychol.* 63 509–537. 10.1111/j.1744-6570.2010.01179.x

[B27] Goncalo J. A. Flynn F. J. Kim S. H. (2010). Are two narcissists better than one? The link between narcissism, perceived creativity, and creative performance. *Pers. Soc. Psychol. Bull.* 36 1484–1495. 10.1177/0146167210385109 20947771

[B28] Grijalva E. Harms P. D. Newman D. A. Gaddis B. H. Fraley R. C. (2015a). Narcissism and leadership: a meta-analytic review of linear and nonlinear relationships. *Pers. Psychol.* 68 1–47. 10.1111/peps.12072

[B29] Grijalva E. Newman D. A. Tay L. Donnellan M. B. Harms P. D. Robins R. W. (2015b). Gender differences in narcissism: a meta-analytic review. *Psychol. Bull.* 141 261–310. 10.1037/a0038231 25546498

[B30] Gudjonsson G. H. Sigurdsson J. F. (2003). The relationship of compliance with coping strategies and self-esteem. *Eur. J. Psychol. Assess.* 19 117–123. 10.1027//1015-5759.19.2.117

[B31] Hall R. J. Snell A. F. Foust M. S. (1999). Item parceling strategies in SEM: investigating the subtle effects of unmodeled secondary constructs. *Organ. Res. Methods* 2 233–256. 10.1177/109442819923002

[B32] Harris K. J. Kacmar K. M. Zivnuska S. (2007). An investigation of abusive supervision as a predictor of performance and the meaning of work as a moderator of the relationship. *Leadersh. Q.* 18 252–263. 10.1016/j.leaqua.2007.03.007

[B33] Harvey S. Keashly L. (2003). Predicting the risk for aggression in the workplace: risk factors, self-esteem and time at work. *Soc. Behav. Pers.* 31 807–814. 10.2224/sbp.2003.31.8.807

[B34] Hayes A. F. (2014). Comparing conditional effects in moderated multiple regression: implementation using process for SPSS and SAS. White paper. Retrieved from http://www.afhayes.com

[B35] Hayes A. F. (2014). *Comparing Conditional Effects in Moderated Multiple Regression: Implementation Using PROCESS for SPSS and SAS. White Paper.* Available at: http://www.afhayes.com

[B36] Hu L. T. Bentler P. M. (1999). Cutoff criteria for fit indexes in covariance structure analysis: conventional criteria versus new alternatives. *Struct. Equ. Modeling* 6 1–55. 10.1080/10705519909540118

[B37] Judge T. A. Bono J. E. (2001). Relationship of core self-evaluations traits—self-esteem, generalized self-efficacy, locus of control, and emotional stability—with job satisfaction and job performance: a meta-analysis. *J. Appl. Psychol.* 86 80–92. 10.1037/0021-9010.86.1.80 11302235

[B38] Judge T. A. Bono J. E. Ilies R. Gerhardt M. W. (2002). Personality and leadership: a qualitative and quantitative review. *J. Appl. Psychol.* 87 765–780. 10.1037/0021-9010.87.4.765 12184579

[B39] Judge T. A. Erez A. Bono J. E. Thoresen C. J. (2003). The core self-evaluations scale: development of a measure. *Pers. Psychol.* 56 303–331. 10.1111/j.1744-6570.2003.tb00152.x

[B40] Judge T. A. Lepine J. A. Rich B. L. (2006). Loving yourself abundantly: relationship of the narcissistic personality to self-and other perceptions of workplace deviance, leadership, and task and contextual performance. *J. Appl. Psychol.* 91 762–776. 10.1037/0021-9010.91.4.762 16834504

[B41] Judge T. A. Piccolo R. F. Kosalka T. (2009). The bright and dark sides of leader traits: a review and theoretical extension of the leader trait paradigm. *Leadersh. Q.* 20 855–875. 10.1016/j.leaqua.2009.09.004

[B42] Kark R. Shamir B. Chen G. (2003). The two faces of transformational leadership: empowerment and dependency. *J. Appl. Psychol.* 88 246–255. 10.1037/0021-9010.88.2.246 12731708

[B43] Kellett J. B. Humphrey R. Sleeth R. G. (2006). Empathy and the emergence of task and relations leaders. *Leadersh. Q.* 17 146–162. 10.1016/j.leaqua.2005.12.003

[B44] Kiazad K. Restubog S. L. D. Zagenczyk T. J. Kiewitz C. Tang R. L. (2010). In pursuit of power: the role of authoritarian leadership in the relationship between supervisors’ Machiavellianism and subordinates’ perceptions of abusive supervisory behavior. *J. Res. Pers.* 44 512–519. 10.1016/j.jrp.2010.06.004

[B45] Krasikova D. V. Green S. G. LeBreton J. M. (2013). Destructive leadership: a theoretical review, integration, and future research agenda. *J. Manag.* 39 1308–1338. 10.1177/0149206312471388

[B46] Leary M. R. Baumeister R. F. (2000). The nature and function of self-esteem: sociometer theory. *Adv. Exp. Soc. Psychol.* 32 1–62. 10.1016/s0065-2601(00)80003-9

[B47] Leckelt M. Küfner A. C. P. Nestler S. Back M. D. (2015). Behavioral processes underlying the decline of narcissists’ popularity over time. *J. Pers. Soc. Psychol.* 109 856–871. 10.1037/pspp0000057 26191958

[B48] Little T. D. Cunningham W. A. Shahar G. Widaman K. F. (2002). To parcel or not to parcel: exploring the question, weighing the merits. *Struct. Equ. Modeling* 9 151–173. 10.1207/s15328007sem0902_1

[B49] Liu J. Kwan H. K. Wu L. Wu W. (2010). Abusive supervision and subordinate supervisor-directed deviance: the moderating role of traditional values and the mediating role of revenge cognitions. *J. Occupat. Organ. Psychol.* 83 835–856. 10.1348/096317909x485216

[B50] Lobbestael J. Baumeister R. F. Fiebig T. Eckel L. A. (2014). The role of grandiose and vulnerable narcissism in self-reported and laboratory aggression and testosterone reactivity. *Pers. Individ. Dif.* 69 22–27. 10.1016/j.paid.2014.05.007

[B51] Luthans F. Peterson S. J. Ibrayeva E. (1998). The potential for the “dark side” of leadership in post communist countries. *J. World Bus.* 33 185–201. 10.1016/s1090-9516(98)90005-0

[B52] Mackey J. D. Frieder R. E. Brees J. R. Martinko M. J. (2015). Abusive supervision: a meta-analysis and empirical review. *J. Manag.* 43 1940–1965. 10.1177/0149206315573997 16093745

[B53] Marsh H. W. Hau K. T. Wen Z. (2004). In search of golden rules: comment on hypothesis-testing approaches to setting cutoff values for fit indexes and dangers in overgeneralizing Hu and Bentler’s (1999) findings. *Struct. Equ. Model.* 11 320–341. 10.1207/s15328007sem1103_2

[B54] Martin S. R. Côté S. Woodruff T. (2016). Echoes of our upbringing: how growing up wealthy or poor relates to narcissism, leader behavior, and leader effectiveness. *Acad. Manag. J.* 59 2157–2177. 10.5465/amj.2015.0680

[B55] Martinez M. A. Zeichner A. Reidy D. E. Miller J. D. (2008). Narcissism and displaced aggression: effects of positive, negative, and delayed feedback. *Pers. Individ. Dif.* 44 140–149. 10.1016/j.paid.2007.07.012

[B56] Martinko M. J. Harvey P. Brees J. R. Mackey J. (2013). A review of abusive supervision research. *J. Organ. Behav.* 34 S120–S137. 10.1002/job.1888

[B57] Matthiesen S. B. Einarsen S. (2001). MMPI-2 configurations among victims of bullying at work. *Eur. J. Work Organ. Psychol.* 10 467–484. 10.1080/13594320143000753

[B58] McClelland G. H. Judd C. M. (1993). Statistical difficulties of detecting interactions and moderator effects. *Psychol. Bull.* 114 376–390. 10.1037/0033-2909.114.2.376 8416037

[B59] McDonald R. P. (1999). *Test Theory: A Unified Treatment.* Mahwah, NJ: Lawrence Erlbaum Associates 10.1111/j.2044-8317.1981.tb00621.x

[B60] Miller J. D. Gentile B. Carter N. T. Crowe M. Hoffman B. J. Campbell W. K. (2017). A comparison of the nomological networks associated with forced-choice and likert formats of the narcissistic personality inventory. *J. Pers. Assess.* 10.1080/00223891.2017.1310731 [Epub ahead of print]. 28436690

[B61] Mitchell M. S. Ambrose M. L. (2007). Abusive supervision and workplace deviance and the moderating effects of negative reciprocity beliefs. *J. Appl. Psychol.* 92 1159–1168. 10.1037/0021-9010.92.4.1159 17638473

[B62] Moon J. H. Lee E. Lee J. A. Choi T. R. Sung Y. (2016). The role of narcissism in self-promotion on instagram. *Pers. Individ. Dif.* 101 22–25. 10.1016/j.paid.2016.05.042

[B63] Nakagawa S. Schielzeth H. (2013). A general and simple method for obtaining R2 from generalized linear mixed-effects models. *Methods Ecol. Evol.* 4 133–142. 10.1111/j.2041-210x.2012.00261.x

[B64] Nevicka B. De Hoogh A. H. B. Van Vianen A. E. M. Beersma B. McIlwain D. (2011a). All I need is a stage to shine: Narcissists’ leader emergence and performance. *Leadersh. Q.* 22 910–925. 10.1016/j.leaqua.2011.07.011

[B65] Nevicka B. Ten Velden F. S. De Hoogh A. H. B. Van Vianen A. E. M. (2011b). Reality at odds with perceptions: narcissistic leaders and group performance. *Psychol. Sci.* 22 1259–1264. 10.1177/0956797611417259 21931153

[B66] Ong C. W. Roberts R. Arthur C. A. Woodman T. Akehurst S. (2016). The leader ship is sinking: a temporal investigation of narcissistic leadership. *J. Pers.* 8 237–247. 10.1111/jopy.12155 25487857

[B67] Orth U. Luciano E. C. (2015). Self-esteem, narcissism, and stressful life events: testing for selection and socialization. *J. Pers. Soc. Psychol.* 109 707–721. 10.1037/pspp0000049 26011661

[B68] Orth U. Robins R. W. Meier L. L. Conger R. D. (2016). Refining the vulnerability model of low self-esteem and depression: disentangling the effects of genuine self-esteem and narcissism. *J. Pers. Soc. Psychol.* 110 133–149. 10.1037/pspp0000038 25915133

[B69] Owens B. P. Wallace A. S. Waldman D. A. (2015). Leader narcissism and follower outcomes: the counterbalancing effect of leader humility. *J. Appl. Psychol.* 100 1203–1214. 10.1037/a0038698 25621592

[B70] Padilla A. Hogan R. Kaiser R. B. (2007). The toxic triangle: destructive leaders, susceptible followers, and conducive environments. *Leadersh. Q.* 18 176–194. 10.1016/j.leaqua.2007.03.001

[B71] Park S. W. Colvin C. R. (2015). Narcissism and other-derogation in the absence of ego threat. *J. Pers.* 83 334–345. 10.1111/jopy.12107 24934570

[B72] Paulhus D. L. (1998). Interpersonal and intrapsychic adaptiveness of trait self enhancement: a mixed blessing? *J. Pers. Soc. Psychol.* 74 1197–1208. 10.1037/0022-3514.74.5.1197 9599439

[B73] Paulhus D. L. Williams K. M. (2002). The dark triad of personality: narcissism, machiavellianism, and psychopathy. *J. Res. Pers.* 36 556–563. 10.1016/S0092-6566(02)00505-6

[B74] Paunonen S. V. Lönnqvist J. E. Verkasalo M. Leikas S. Nissinen V. (2006). Narcissism and emergent leadership in military cadets. *Leadersh. Q.* 17 475–486. 10.1016/j.leaqua.2006.06.003

[B75] Pearce J. L. Porter L. W. (1986). Employee responses to formal performance appraisal feedback. *J. Appl. Psychol.* 71 211–218. 10.1037/0021-9010.71.2.211

[B76] Penney L. M. Spector P. E. (2002). Narcissism and counterproductive work behavior: do bigger egos mean bigger problems? *Int. J. Select. Assess.* 10 126–134. 10.1111/1468-2389.00199

[B77] Peterson S. J. Galvin B. M. Lange D. (2012). CEO servant leadership: exploring executive characteristics and firm performance. *Pers. Psychol.* 65 565–596. 10.1111/j.1744-6570.2012.01253.x

[B78] Preacher K. J. Hayes A. F. (2004). SPSS and SAS procedures for estimating indirect effects in simple mediation models. *Behav. Res. Methods* 36 717–731. 10.3758/bf03206553 15641418

[B79] Preacher K. J. Rucker D. D. Hayes A. F. (2007). Addressing moderated mediation hypotheses: theory, methods, and prescriptions. *Multivariate Behav. Res.* 42 185–227. 10.1080/00273170701341316 26821081

[B80] Raskin R. Hall C. S. (1981). The narcissistic personality inventory: alternative form reliability and further evidence of construct validity. *J. Pers. Assess.* 45 159–162. 10.1207/s15327752jpa4502_10 16370732

[B81] Raskin R. N. Hall C. S. (1979). A narcissistic personality inventory. *Psychol. Rep.* 45:590 10.1037/t00001-000 538183

[B82] Raykov T. Dimitrov D. M. Asparouhov T. (2010). Evaluation of scale reliability with binary measures using latent variable modeling. *Struct. Equ. Modeling* 17 265–279. 10.1177/0748175609344096

[B83] Rosenberg M. (1965). *Society and the Adolescent Self-Image.* Princeton, NJ: Princeton university press 10.1515/9781400876136

[B84] Schaufeli W. B. Leiter M. P. Maslach C. Jackson S. E. (1996). *MBI-General Survey.* Palo Alto, CA: Consulting Psychologists Press.

[B85] Schaufeli W. B. van Dierendonck D. (2000). *Utrechtse Burnout Schaal: Handleiding.* Lisse: Swets Test Publishers.

[B86] Schermelleh-Engel K. Moosbrugger H. Müller H. (2003). Evaluating the fit of structural equation models: tests of significance and descriptive goodness-of-fit measures. *Methods Psychol. Res. Online* 8 23–74.

[B87] Schyns B. Schilling J. (2013). How bad are the effects of bad leaders? A meta-analysis of destructive leadership and its outcomes. *Leadersh. Q.* 24 138–158. 10.1016/j.leaqua.2012.09.001

[B88] Sedikides C. Campbell W. K. (2017). Narcissistic force meets systemic resistance: the energy clash model. *Perspect. Psychol. Sci.* 12 400–421. 10.1177/1745691617692105 28544862

[B89] Shrauger J. S. (1975). Responses to evaluation as a function of initial self-perceptions. *Psychol. Bull.* 82 581–596. 10.1037/h0076791 1099604

[B90] Siemsen E. Roth A. Oliveira P. (2010). Common method bias in regression models with linear, quadratic, and interaction effects. *Organ. Res. Methods* 13 456–476. 10.1177/1094428109351241

[B91] Smith J. A. Foti R. J. (1998). A pattern approach to the study of leader emergence. *Leadersh. Q.* 9 147–160. 10.1016/S1048-9843(98)90002-9

[B92] Smith S. M. Petty R. E. (1995). Personality moderators of mood congruency effects on cognition: the role of self-esteem and negative mood regulation. *J. Pers. Soc. Psychol.* 68 1092–1107. 10.1037/0022-3514.68.6.1092 7608856

[B93] Snijders T. A. Bosker R. J. (1994). Modeled variance in two-level models. *Sociol. Methods Res.* 22 342–363. 10.1177/0049124194022003004

[B94] Soyer R. B. Rovenpor J. L. Kopelman R. E. (1999). Narcissism and achievement motivation as related to three facets of the sales role: attraction, satisfaction and performance. *J. Bus. Psychol.* 14 285–304. 10.1023/A:1022147326001

[B95] Stoeber J. Sherry S. B. Nealis L. J. (2015). Multidimensional perfectionism and narcissism: grandiose or vulnerable? *Pers. Individ. Dif.* 80 85–90. 10.1016/j.paid.2015.02.027

[B96] Stucke T. S. (2003). Who’s to blame? Narcissism and self-serving attributions following feedback. *Eur. J. Pers.* 17 465–478. 10.1002/per.497

[B97] Tepper B. J. (2000). Consequences of abusive supervision. *Acad. Manag. J.* 43 178–190. 10.2307/1556375

[B98] Tepper B. J. (2007). Abusive supervision in work organizations: review, synthesis, and research agenda. *J. Manag.* 33 261–289. 10.1177/0149206307300812

[B99] Tepper B. J. Carr J. C. Breaux D. M. Geider S. Hu C. Hua W. (2009). Abusive supervision, intentions to quit, and employees’ workplace deviance: a power/dependence analysis. *Organ. Behav. Hum. Decis. Process.* 109 156–167. 10.1016/j.obhdp.2009.03.004

[B100] Tepper B. J. Henle C. A. Lambert L. S. Giacalone R. A. Duffy M. K. (2008). Abusive supervision and subordinates’ organization deviance. *J. Appl. Psychol.* 93 721–732. 10.1037/0021-9010.93.4.721 18642979

[B101] Thoroughgood C. N. Padilla A. Hunter S. T. Tate B. W. (2012). The susceptible circle: a taxonomy of followers associated with destructive leadership. *Leadersh. Q.* 23 897–917. 10.1016/j.leaqua.2012.05.007

[B102] Twenge J. M. Foster J. D. (2010). Birth cohort increases in narcissistic personality traits among American college students, 1982-2009. *Soc. Psychol. Pers. Sci.* 1 99–106. 10.1177/1948550609355719

[B103] Twenge J. M. Konrath S. Foster J. D. Campbell W. K. Bushman B. J. (2008). Egos inflating over time: a cross-temporal meta-analysis of the narcissistic personality inventory. *J. Pers.* 76 875–902. 10.1111/j.1467-6494.2008.00507.x 18507710

[B104] Watts A. L. Lilienfeld S. O. Smith S. F. Miller J. D. Campbell W. K. Waldman I. D. (2013). The double-edged sword of grandiose narcissism: implications for successful and unsuccessful leadership among U.S. Presidents. *Psychol. Sci.* 24 2379–2389. 10.1177/0956797613491970 24104503

[B105] West P. Sweeting H. (1997). “Lost souls” and “rebels”: a challenge to the assumption that low self-esteem and unhealthy lifestyles are related. *Health Educ.* 97 161–167. 10.1108/09654289710172450

[B106] Widaman K. F. Little T. D. Preacher K. J. Sawalani G. M. (2011). “On creating and using short forms of scales in secondary research,” in *Secondary Data Analysis: An Introduction for Psychologists* eds Trzesniewski K. H. Donnellan M. B. Lucas R. E. (Washington, DC: American Psychological Association) 39–62. 10.1037/12350-003

[B107] Wisse B. Sleebos E. (2016). When the dark ones gain power: perceived position power strengthens the effect of supervisor Machiavellianism on abusive supervision in work teams. *Pers. Individ. Dif.* 99 122–126. 10.1016/j.paid.2016.05.019

[B108] Wu T. Y. Hu C. (2009). Abusive supervision and employee emotional exhaustion: Dispositional antecedents and boundaries. *Group Organ. Manag.* 34 143–169. 10.1177/1059601108331217

[B109] Xiaqi D. Kun T. Chongsen Y. Sufang G. (2012). Abusive supervision and LMX: leaders’ emotional intelligence as antecedent variable and trust as consequence variable. *Chin. Manag. Stud.* 6 257–270. 10.1108/17506141211236695

[B110] Xu E. Huang X. Lam C. K. Miao Q. (2012). Abusive supervision and work behaviors: the mediating role of LMX. *J. Organ. Behav.* 33 531–543. 10.1002/job.768

[B111] Zapf D. (1999). Organisational, work group related and personal causes of mobbing/bullying at work. *Int. J. Manpower* 20 70–85. 10.1108/01437729910268669

[B112] Zellars K. L. Tepper B. J. Duffy M. K. (2002). Abusive supervision and subordinates’ organizational citizenship behavior. *J. Appl. Psychol.* 87 1068–1076. 10.1037//0021-9010.87.6.1068 12558214

